# Editorial: Novel diagnostic and prognostic methods in acute kidney injury among patients in intensive care unit

**DOI:** 10.3389/fneph.2025.1586794

**Published:** 2025-03-20

**Authors:** Marco Fiorentino

**Affiliations:** Department of Precision and Regenerative Medicine and Ionian Area, University of Bari Aldo Moro, Bari, Italy

**Keywords:** acute kidney injury, biomarkers, critical care, omics, artificial intelligence

Acute Kidney Injury (AKI) remains a significant challenge in intensive care units (ICUs), contributing to a higher rate of morbidity and mortality globally (1). Several clinical conditions, such as sepsis, the use of nephrotoxic drugs, or hemodynamic instability, may cause this abrupt decline in kidney function (2). Early diagnosis and accurate prognosis of AKI are critical in improving patient short- and long-term outcomes; in this scenario, the development and implementation of novel diagnostic and prognostic methods may help clinicians to prevent or treat AKI, reducing long-term sequels (3).

Traditional diagnostic criteria for AKI, such as serum creatinine levels and urine output, are late indicators of renal dysfunction, and their increase typically occurred in the latest phase of the disease process, delaying therefore clinical diagnosis and potential intervention (4). More sensitive and specific biomarkers have been recently introduced in the last decades, with the aim of facilitating AKI prediction or early detection among critically ill patients. Among them, neutrophil gelatinase-associated lipocalin (NGAL), kidney injury molecule-1 (KIM-1), and tissue inhibitor of metalloproteinases-2 (TIMP-2) combined with insulin-like growth factor-binding protein 7 (IGFBP7) have shown promise as biomarkers for early AKI detection in different settings, including sepsis, drug toxicity, cardiac-surgery AKI, in both preclinical and clinical models, with controversial results (4). Recently, a biomarker-guided approach has shown to be effective in initiating timely interventions, potentially mitigating renal damage and improving patient outcomes (5). In this Research Topic, Nusshag et al. analyzed the diagnostic value of [TIMP-2]*[IGFBP7] and soluble urokinase plasminogen activator receptor (suPAR) in predicting post-operative KDIGO stage 2-3 AKI among 75 patients undergoing elective aortic surgery (6); although the limited sample size may hamper clinical significance, both of these markers were not superior in terms of diagnostic accuracy to standard parameters (cystatin C, serum creatinine, urine output). In addition, the inability of the kidneys to excrete sodium may represent a typical hallmark of critically ill patients who are going to develop AK, as urinary sodium excretion was shown to be low one day before AKI onset due to the high expression of Na+/H+ exchanger at proximal tubular level (Morais et al.).

In the next future, the integration of novel approaches, such as real-time monitoring systems and point-of-care testing, may represent a step forward to AKI detection capabilities. New imaging modalities, such as contrast-enhanced ultrasound and magnetic resonance imaging (MRI), may provide further non-invasive insights into renal perfusion and structural integrity, potentially identifying kidney damage in an earlier phase (6). Additionally, advancements in biosensor technologies and microfluidics have improved real-time AKI monitoring, enabling more precise and timely assessments in critically ill patients (7).

Beyond diagnosis, accurately predicting the progression and outcomes of AKI remains a key focus in critical care patients. AKI prognosis depends on various factors, including age, comorbidities, baseline kidney function, renal functional reserve, AKI etiology, severity, duration, and treatment-related factors (timely AKI identification and intervention, fluid management, avoidance of nephrotoxic drugs). In this scenario, several studies have also demonstrated the potential accuracy of some plasma and urine biomarkers in predicting renal recovery after AKI (8). Moreover, fibrinogen levels are typically altered during sepsis and correlated with organ dysfunction and mortality even in the context of AKI patients. In this Research Topic, Chen et al. investigated the potential role of fibrinogen as a prognostic marker for sepsis-associated AKI, highlighting a nonlinear relationship between fibrinogen levels and 28-day mortality, with a reduction in mortality as fibrinogen increased in patients when fibrinogen levels was below 1.6 g/L.

In addition, artificial intelligence (AI) and machine learning algorithms have demonstrated considerable potential in predicting AKI identification and patients’ stratification in different clusters of progression, analyzing all the data including in the electronic health records (9). These AI-driven models offer personalized risk stratification, allowing for tailored therapeutic strategies.

Additionally, the integration of omics technologies, including genomics, transcriptomics, proteomics, metabolomics and epigenomics, is going to revolutionize our knowledge into the molecular and cellular mechanisms underlying AKI (10). Similar to what already reported in several kidney diseases, such as in several glomerulonephritis, the evaluation of genetic polymorphisms related to renal injury susceptibility and metabolic profiling of kidney function alterations may provide crucial insights into the understanding of disease trajectories, enhancing precision medicine in AKI management.

With advancements in diagnosis and prognosis, the potential effects of therapeutic interventions are also being explored to mitigate and potentially reverse AKI severity. Although sodium bicarbonate infusion represents a typical treatment for metabolic acidosis in patients with kidney dysfunction, its correlation to hospital mortality among AKI patients is still not well investigated. In this Research Topic, Wang et al. reported data from 390 patients selected from the MIMIC-IV database after a propensity score matching approach, describing the potential beneficial effects of such treatment on hospital mortality only in AKI patients with high anion gap metabolic acidosis. In the last decade, novel pharmacological innovations have been investigated for their nephroprotective effects, and novel drug delivery systems, including nanomedicine-based approaches and cell-based therapies, may enhance the precision and efficacy of AKI treatments, reducing inflammation and promoting renal repair. Extracellular vesicles derived from stem cells have also demonstrated regenerative potential in preclinical models. Finally, renal replacement therapies should be personalized and tailored based to the specific setting and patient’s conditions. In this Research Topic, an interesting case report reported the beneficial effects of a timely treatment with the molecular adsorber recirculating system (MARS) in AKI patients affecting by bile cast nephropathy (Issac et al.).

In summary, the landscape of AKI diagnosis and prognosis in ICU settings is rapidly evolving, driven by advancements in biomarkers, imaging techniques, artificial intelligence, omics technologies and emerging therapeutics. However, several challenges persist in widely implementing these novel diagnostic and prognostic methods in ICUs, including the high cost of biomarker assays and imaging technologies, the standardization of biomarker thresholds and the validation of AI-based models across different AKI phenotypes. In the next future, AKI research should focus on multicenter studies with a multidisciplinary collaboration (nephrologists, intensivists, statisticians, epidemiologists) in order to refine and validate these novel approaches. By embracing cutting-edge technologies and fostering collaborative research efforts, the medical community can pave the way for precision medicine in AKI management in critically ill patients.
